# Application of the extended theory of planned behavior in predicting the behavioral intentions of Iranian local communities toward forest conservation

**DOI:** 10.3389/fpsyg.2023.1121396

**Published:** 2023-01-19

**Authors:** Moslem Savari, Bagher Khaleghi

**Affiliations:** ^1^Department of Agricultural Extension and Education, Agricultural Sciences and Natural Resources University of Khuzestan, Mollasani, Iran; ^2^Department of Forestry Policy and Economic, University of Tehran, Karaj, Iran

**Keywords:** forest protection, local communities, risk perception, theory of planned behavior, Iran

## Abstract

Natural forests are the habitat of many plant and animal species and are the main source of genetic reserves. In addition to preserving biodiversity, forests play an important role in the livelihood and income of many indigenous communities. But, in the last few decades, due to the lack of proper management of the beneficiaries, they have been exposed to destruction, so that their protection requires the participation of all members of the society, especially the local people. Therefore, the main goal of this research was to discover the determinant factors on the intention of local communities to protect forests in Iran. An extended theory of planned behavior (TPB) by adding the variables of “risk perception” (RP) and “sense of belonging to place” (SBP) was used as the theoretical framework of the research. This research was done using the questionnaire survey method and with the help of structural equation modeling (SEM). The statistical population of this study included all the rural communities living in the Arasbaran forests (located in the northwest of Iran). The research findings indicated that the original TPB explains 66.9% of the variance of the behavioral intentions of locals toward forest protection. The three main constructs of the original TPB included attitude, perceived behavioral control (PBC) and subjective norms (SN), all of which had positive effects on the intention of local communities. Most importantly, the extended TPB by including RP and SBP increases the ability of the model to explain the intentions of local communities to forest protection by 11.8%. In this study, the variable of RP was identified as the most important factor, so it is suggested to the policy-makers of this field to increase the RP of local communities in relation to forest destruction using communication media. It is also suggested to promote safe behaviors in these natural areas through developing forest protection organizations and properly training locals on the protection of forest areas.

## Introduction

The high rate of species extinction and the inevitable human impacts on biodiversity have increased the need to preserve, restore and sustainably use of ecosystems (Oettel and Lapin, 2021; Valizadeh et al., 2021a; Savari et al., 2022d). Natural resources, especially natural forests, are under increasing pressure worldwide due to economic and population growth as well as related changes in different consumption habits (Abdulkarim et al., 2017; Scholes et al., 2018; Valizadeh et al., 2021b; Savari, 2023). Forests play an important role in the stability of the Earth’s biological processes (Wang et al., 2019) and provide many direct and indirect economic benefits to forest-dependent communities in the world, especially in developing countries (Jannat et al., 2020; Rosmanita et al., 2021). These resources play a vital role in preserving natural resources, they protect water and soil and regulate climate, and most importantly, are a barrier against desertification (Ahmed et al., 2020). Forests cover a large part of the Earth’s biodiversity (Kok et al., 2017), which it leads to providing a wide range of benefits and services to society (Ahmed et al., 2020). For example, forests are essential in ensuring access to water because they produce the highest quality of water of any ecosystem, which is crucial for ecological needs and survival of human (Empidi and Emang, 2021). Forests also produce different types of food, fuel, fiber, etc. for millions of people around the world. Therefore, they are the source of providing economic benefits such as livelihood, income and employment on a local, regional and global scale (Newton et al., 2016; Savari et al., 2020). In total, it can be concluded that the world’s forests provide potential contributions to human life in different ways, however, they are still threatened by the expansion of agriculture and other land uses (Carter et al., 2017; Börner et al., 2020; Savari and Zhoolideh, 2021; Savari and Moradi, 2022). Land use change itself is the cause for 27% of global forest destructions (Curtis et al., 2018). Also, various factors such as over-exploitation, livestock grazing, fire and wood harvesting threaten the stability of these ecosystems, so, day by day, the number and quality of plant and animal species in forests are decreasing (Coulibaly-Lingani et al., 2011; Empidi and Emang, 2021). In addition, the people who live inside or at a close distance from the forests are at a low level in terms of welfare, services, health and education facilities, and are highly dependent on these resources for their livelihood and other needs. This has caused them to destroy forests directly or indirectly and knowingly or unknowingly (Jana et al., 2014). Therefore, even if events such as fires or storms naturally cause forest destruction, however, for the above-mentioned reasons, disturbances caused by human activities are often at the center of deforestation analyzes (Börjeson and Ango, 2021). Currently, in most rural areas of Iran, local people are in close relationship with their surrounding natural environment, especially forests, and a large part of their lives depends on the forest products (Savari et al., 2022b). This excessive dependence of livelihood, economic benefits and income of rural communities on forests around the world has had a great impact on their behavior with these resources (Jannat et al., 2020). Therefore, in order to preventing the destruction of forests, it is necessary to make a comprehensive and collective change in the direction of sustainable behavior (Schneiderhan-Opel and Bogner, 2021).

For decades, researchers have been searching for variables that influence behavior and identifying those that have the greatest impact on it (Strydom, 2018). TPB provides more insight into the prediction of human’s behavior than other socio-economic theories (Lam, 2006; Greaves et al., 2013) and is one of the most popular social-psychological models for understanding and predicting human behavior (Ajzen, 2015; Soorani and Ahmadvand, 2019). This theory states that actual behavior can be better predicted by behavioral intention (Popa et al., 2019). The intention variable is an excellent predictor of actual environmental behaviors (Holt et al., 2021). In TPB, behavioral intention is the best direct determinant of actual behavior (Empidi and Emang, 2021) and the closest predictor of behavior (Sánchez et al., 2018). Therefore, in this study, TPB was utilized for predicting the behavioral intentions of local communities in the conservation of Arasbaran forests. In this regard, the goals of this research include (i) investigating the behavioral intentions of local communities towards participation in forest protection (ii) investigating the power of TPB in explaining the behavioral intentions of locals towards participation in forest protection (iii) extending the TPB model to increase its explanatory power and (iv) providing practical and beneficial policies to assist policy-makers and decision-makers in the field of forest and natural resources.

## Theory of planned behavior

This socio-psychological theory states that the actual behavior can be predicted by the behavioral intention (Popa et al., 2019; Tam, 2020; Gholamrezai et al., 2021). According to TPB, intentions, which reflect the motivation or plan for engaging in an action, are the strongest predictors of behavior (Sánchez et al., 2018; Zhong et al., 2019). Attitude plays a central role in the TPB (Zhong et al., 2019) because the attitude toward a behavior indicates the context in which a person has a favorable or unfavorable evaluation of that behavior (Sánchez et al., 2018; Ullah et al., 2021). For the formation of pro-environmental behaviors (such as forest conservation), a positive attitude must be created in this regard (Holt et al., 2021; Ullah et al., 2021; Karimi and Ataei, 2022); therefore, people’s attitude towards forest protection can contribute to its sustainability (Steel, 1981; Meijer et al., 2015; Koop et al., 2019; Farani et al., 2021).

Another variable of this theory is subjective norm (SN) or social pressure. SN refers to the pressure or social influence of individuals when faced with a behavioral choice (Sánchez et al., 2018). In addition, SN originates from the influence of the behavior and words of some important individuals in a person’s life (Gao et al., 2017) and is a person’s perceptual belief about whether others support his behavior change or not (Liang et al., 2018). The construct of perceived behavioral control (PBC) as the third determining factor of behavioral intention is related to the person’s understanding from the ease and difficulty of performing a behavior (Empidi and Emang, 2021). In other words, PBC refers to the perceived ease or difficulty and finally performing a specific behavior (Sánchez et al., 2018; Empidi and Emang, 2021). PBC includes two aspects: 1) The level of control of a person over a behavior 2) The level of self-confidence of a person about his ability for conducting or not conducting that behavior (Empidi and Emang, 2021). In the following, numerous studies related to forestry that were done based on the TPB were examined.

Karppinen (2005) in a study in Finland showed that the choice of reforestation method can be described by the TPB, in this study the results indicated that attitude is the strongest explanatory factor and SN and PBC clearly had smaller but equally reciprocal effects on reforestation intention. Primmer and Karppinen (2010) in a research using TPB emphasized the significant effect of foresters’ attitude and SN on the protection of biodiversity. Karppinen and Berghäll (2015) in Finland using this theory studied the intention for take forest improvement measures, their results showed that the SN was the most important explanatory factor and attitudes had less explanatory power. In the research of Castilho et al. (2018), who investigated the attitude and behavior of rural residents towards different motives for hunting and deforestation in protected areas of Brazil, their findings showed that management measures to change the behavior of the residents towards the protection of the forests of the study area should consider the attitudes and norms of the people. Popa et al. (2019) in a study used TPB for intentions to engage in forest law enforcement in Romania. The results showed that the intention to this engagement is predicted by attitude, SN and PBC. Empidi and Emang (2021) in their study in Malaysia tried to understand the public intentions for participating in protection initiatives for forested watershed areas using the TPB. The findings indicated that attitude significantly affects people’s behavioral intention. This indicates the importance of creating the conditions to encourage people’s behavioral intention towards conservation initiatives that ultimately leads to forest sustainability. Therefore, the three main hypotheses of this theory were formed as follows:

*Hypothesis 1*: The attitude of local communities affects their intention for protecting the forest.

*Hypothesis 2*: SN affects the intention of local communities for protecting the forest.

*Hypothesis 3*: PBC affects the intention of local communities for protecting the forest.

### The extended theory of planned behavior

Ajzen, as one of the founders of this type of behavior investigation, states that in order to improve this theory, new variables and components can be considered (Ajzen, 2005). Therefore, many researchers have studied other variables that are not included in this framework and believe that adding them can improve the prediction power of the model (Yadav and Pathak, 2016; Bird et al., 2018; Rezaei et al., 2019). Although TPB has been widely used to test the relationship between attitudinal structures and behavioral intention with great success, however, it is not a comprehensive model and neglected the impact of influencing variables such as RP (Bagheri et al., 2019; Savari and Gharechaee, 2020).

RP was the second important variable added to TPB. RP has been well determined in pro-environmental behaviors (Savari and Gharechaee, 2020; Li et al., 2021; Megías-Robles et al., 2022). Previous studies have clearly argued that RP is a strong variable in explaining behavioral intentions (Savari and Gharechaee, 2020). In fact, understanding of environmental risks can improve people’s pro-environmental behavior, which is one of the important factors in protecting the environment (Sabzehei et al., 2016). RP is actually a subjective belief (whether rational or irrational) about the chance of a risk occurring or about the time occurring and the consequences of that risk by an individual, group or society (Mumpower et al., 2016). Therefore, RP can change the result of many environmental activities (Bagheri et al., 2019; Aliabadi et al., 2020). For example, Savari and Gharechaee (2020) in a research concluded that farmers who had a high RP toward chemical fertilizers were more inclined to use chemical fertilizers safely. Therefore, the hypothesis of this section is presented as follows.

*Hypothesis 4*: RP by local communities affects their intention for protecting the forest.

**Figure 1:**
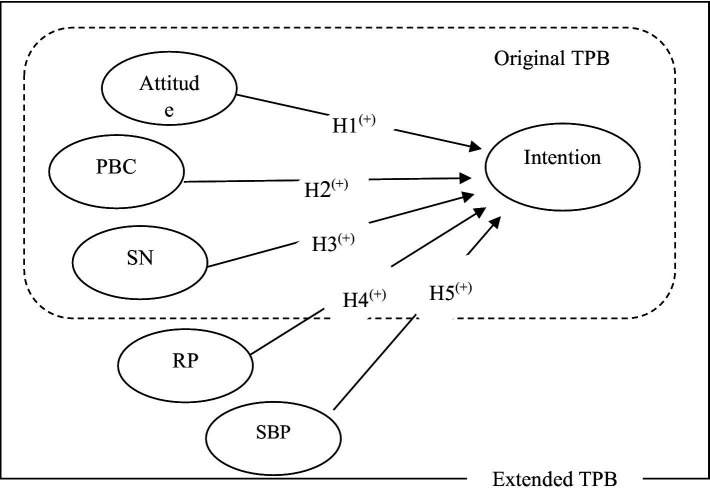
Research framework in this research.

SBP is another factor influencing the implementation of forest protection behavior. This factor can be described as an emotional and meaningful relationship between a person and a place (Le Dang et al., 2014). It expresses people’s positive beliefs and feelings about the environment, so that people with higher SBP will use more protective behavior (Milligan, 1998). SBP refers to the cognitive relationship of an individual or a group with an environment, and in terms of identity, this factor is the relationship of belonging and identity of an individual to the social environment in which he lives (Lee and Shen, 2013). This sense is higher than the sense of place and it has a decisive role in the continuation of human presence in the environment and its protection (Le Dang et al., 2014), because this feeling leads to the connection of the person with the place, and in that human considers himself as a part of the place and respects it a lot (Elias et al., 2018). For example, Savari et al. (2021) stated that SBP can improve the environmental attitude of rural women in forest protection and make them adopt protective behaviors. In a research in this field, Zwiers et al. (2018) also showed that place values and attachments have a significant effect in predicting safe and pro-environmental behaviors (Figure 1).

*Hypothesis 5*: Local communities SBP affects their intention for protecting the forest.

## Materials and methods

### Study area

**Figure 2:**
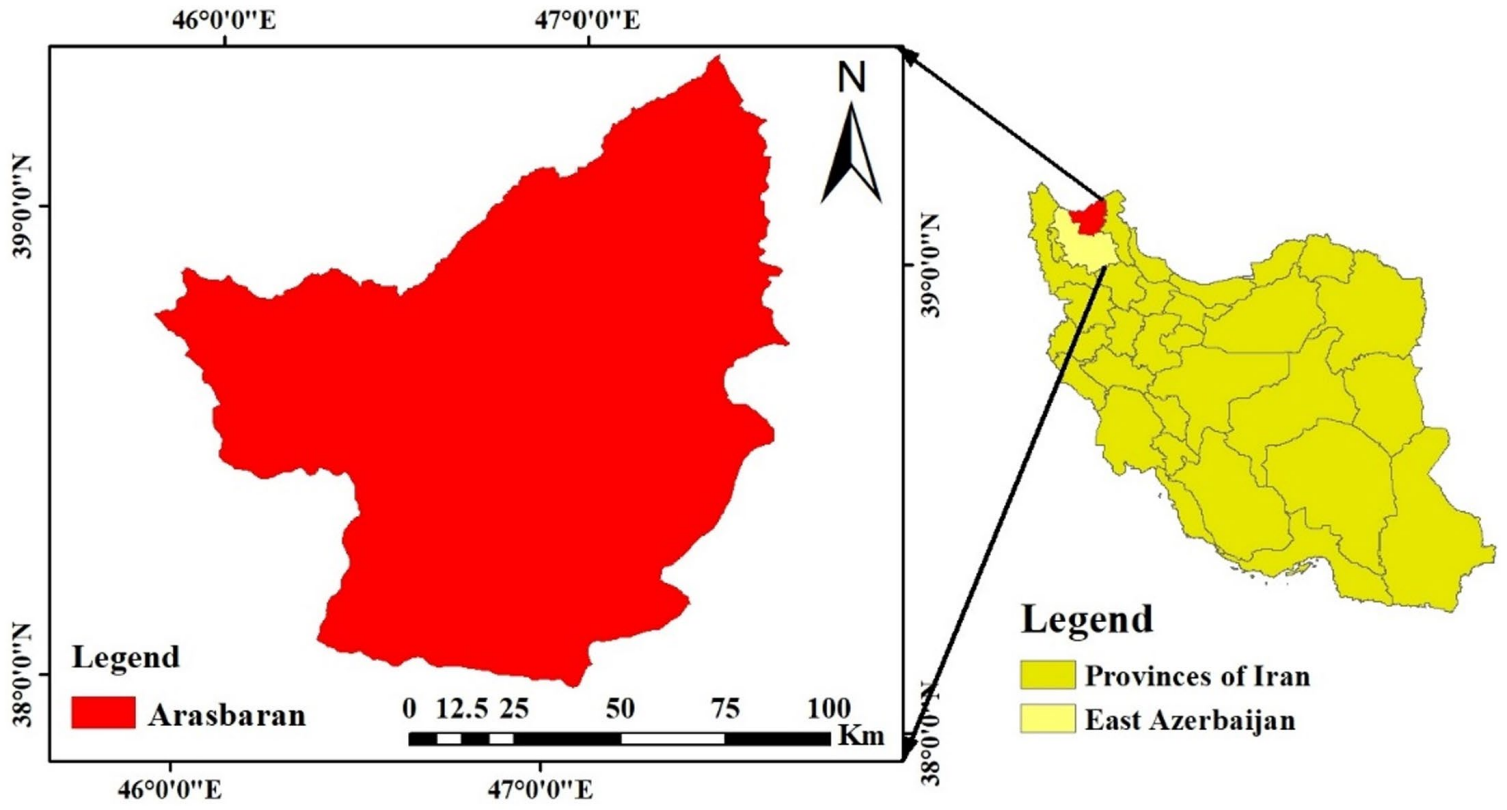
Stuay area.

Arasbaran forest area is located in northwestern part of Iran and covers about 148,000 hectares. Part of the area is listed as one of UNESCO biosphere reserve and national park of Arasbaran since 1976, thus, nominated as UNESCO world heritage site. The area is covered by around 1,072 plant species and 360 animal species (Shirvany, and Hamzeh’ee, 2017). In Arasbaran, there are valuable woody and herbal species. Most important forest trees include *Carpinus betulus* L., *Quercus macranthera* Fisch. and C. A. Mey., *Cornus mas* L., *Crataegus meyeri* A. Pojark., *Prunus spinosa* L., *Acer campestre* L. and *Viburnum lantana* L. (Shirvany, and Hamzeh’ee, 2017). There are also medicinal herb species such as *Origanum vulgare* L., *Cichorium intybus* L., *Echium vulgare* L., *Melilotus officinalis* L., *Malva sylvestris* L., *Satureja laxiflora* C. Koch, *Hyoscyamus niger* L., *Echium italicum* L., and several others that are valuable to humans (Khaleghi et al., 2016). *Trifolium resupinatum* L., *Onobrychis sativa* L., *Artemisia fragrans* Willd., *Agropyron tenerum* Vasey, *Festuca* spp., *Medicago* spp., and others are valuable to grazing livestock. It is also home to various animals including leopard, brown bears, wild boar, jackal, partridge, Armenian ram, Caucasian black grouse, etc. (Shirvany, and Hamzeh’ee, 2017; Figure 2).

### Statistical population and sample size

The statistical population of this study included all people over 15 years old in the rural communities of Arasbaran forests in East Azarbaijan province (located in the northwest of Iran). Using the table of Krejcie and Morgan (1970), the sample size was determined 402 individual. First, due to the spread of the statistical population throughout East Azarbaijan Province, sampling in this research was done using multistage stratified clustered method. Arasbaran forests cover parts of several counties of East Azarbaijan province. First, the districts of these counties that are covered by Arasbaran forests were selected, in the next step, several forest rural districts were chosen from each of these districts, and finally, four villages were selected from each rural district.

### Participation

The examination of the individual characteristics of local communities showed that their average age was 41.52 with a standard deviation of 13.18 years and the studied people were in the age range of 15 to 74 years. In addition, the results of the respondents’ gender showed that 61% were male and 39% were female. The average annual income of the household was 190.85 million Rials per year with a standard deviation of 9.52 million Rials. Also, the results showed that the average number of household members was 4.33 with a standard deviation of 1.17. The findings of the study on the education of local communities indicated that the majority of them, i.e., 28.28%, had diploma and 71.69% of the respondents were not members of forest protection cooperatives.

### Survey instrument

The data of this study was collected in 2022. The main tool was a questionnaire that consisted of two main sections: 1) demographic variables that included age, gender, education level, income, marital status, and membership in forest protection cooperatives; 2) A questionnaire that included 4 items for attitude, 4 items for SN, 4 items for PBC, 5 items for RP, 4 items for intention and 6 items for SBP (Table 1).

**Table 1:** Measurement variables of the research.

Construct	Measurement items	Source
Attitude	Applying forest protection methods by local communities is wise.	Ajzen (2005, 2015), Castilho et al. (2018), Empidi and Emang (2021)
	Applying forest protection methods by local communities is important.	
	Applying forest protection methods by local communities is necessary.	
	Applying forest protection methods by local communities is useful.	
SN	If I protect the forest, my friends, relatives and neighbors will approve my work	Karppinen and Berghäll (2015), Ataei et al. (2022), Savari et al. (2022c), Zobeidi et al. (2022)
	I feel the desire and need of the society to protect the forest.	
	If I protect the forests of Arasbaran, the society will approve my work	
	People whose opinions are important to me ask me to protect forests.	
PBC	I have the necessary physical and financial resources to implement forest protection operations.	Sánchez et al. (2018), Empidi and Emang (2021)
	I have the necessary knowledge and skills to implement forest protection operations.	
	I am sure that if I want, I can implement the forest protection operations.	
	I know how to should protect forests.	
RP	Deforestation rate has increased every year and I have to do something to protect it.	Bagheri et al. (2019), Savari and Gharechaee (2020), Li et al. (2021), Savari et al. (2022b)
	Destruction of forests causes the loss of plant and animal species.	
	Destruction of forests destroys the environment.	
	In the long run, the destruction of forests has negative effects on the livelihood of local communities.	
	Destruction of forests increases soil erosion.	
SBP	There are many emotional relationships between local communities and forests.	Hernandez et al. (2010), Savari et al. (2013, 2015), Morse and Mudgett (2017)
	I have a great attachment to the natural environment and forests of Arasbaran	
	I do not replace living around Arasbaran forests with any other environment.	
	Arasbaran forests have increased my attachment and enthusiasm for living in these areas	
	I feel completely satisfied living around the forests of Arasbaran.	
	I am proud due to living around the forests of Arasbaran.	
Intention	I have intention to protect the environment of forests.	Sánchez et al. (2018), Bagheri et al. (2019), Savari et al. (2022a), Yazdanpanah et al. (2022)
	I like to protect forests for the health of society and the environment	
	I have intention to protect forests for conserving plant and animal species	
	I plan to always protect the forests in order to preserve livelihood of myself and local communities.	

### Reliability and validity

For evaluating the overall validity of the indicators, the survey draft and questions were assessed by an expert group before entering the interview phase with local communities. This expert group included professors of agricultural extension and education, environment, psychology, social sciences, and agricultural sciences. In addition, in this study, the average extracted variance index (AVE) was also utilized. Also, to determine the reliability of the questionnaire, Cronbach’s alpha coefficient and composite reliability (CR) were estimated (Table 2).

**Table 2:** The status of research variables.

Variables	Mean	Sd
Attitude	2.85	0.742
SN	3.14	0.635
PBC	2.42	0.701
RP	2.69	0.655
SBP	3.08	0.827
Intention	2.58	0.677

### Data analysis

In this study, SPSS and Smart PLS software were used to analyze the data in two sections of descriptive and inferential statistics. SEM allows the researcher to statistically model and test complex phenomena (Hair et al., 2017). SEM techniques are methods for confirming or rejecting theoretical models in a quantitative way (Khoshmaram et al., 2020). PLS is the third generation of SEM, which is a suitable method for investigating the relationships between latent variables that are measured by observed variables (Hair et al., 2017). One of the most important reasons for using this method by researchers is that SEM is a complete and comprehensive method for testing the theories of a study (Hair et al., 2014).

#### Ethical statement

**Figure 3:**
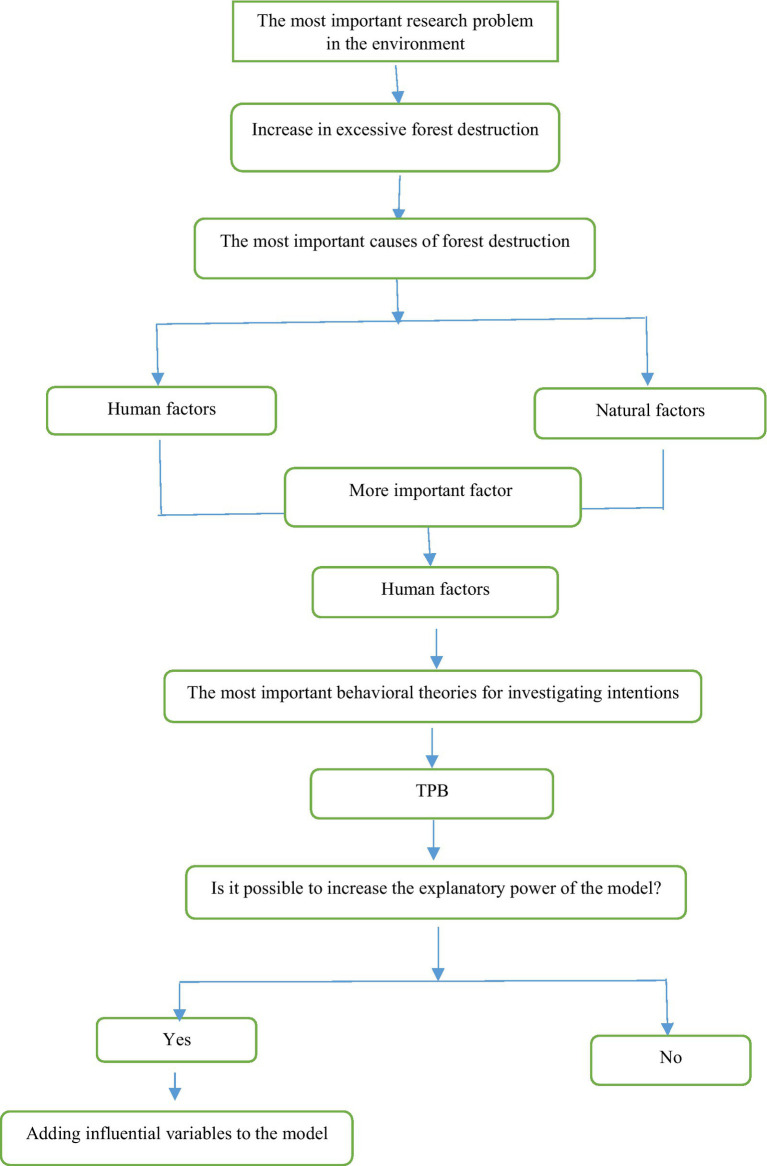
Flowchart of steps this study.

All interviewees were informed about data protection issues by the enumerators and gave their consent orally at the beginning of each interview. Informed consent was obtained from all individual participants included in the study. All materials and methods are performed in accordance with the instructions and regulations and this research has been approved by a committee at Agricultural Sciences and Natural Resources University of Khuzestan, Mollasani, Iran. All procedures performed in studies involving human participants were in accordance with the ethical standards of the institutional research committee and with the 1964 Helsinki declaration and its later amendments or comparable ethical standards (Figure 3).

## Results

### Status of research variables

The findings of examining the status of the extended TPB variables indicate that only two variables, including SN and SBP, are above the average level (3 theoretical medians). The results of other variables show that the locals in the studied area do not have appropriate attitude and intention towards participation in forest protection, in addition, they have low PBC, which requires the development of training forest protection methods among them (Table 2).

### Measurement models

In this section, first-order confirmatory factor analysis (CFA) was used to check the fit of two measurement models (initial and extended). The results of this section are presented in three stages: unidimensionality; reliability and validity; and discriminant validity.

#### Unidimensionality

This step is evaluated based on the factor load and t values. According to the figures in Table 3, it can be said that the factor load values presented for the selected markers (above 0.6) were statistically significant at 1% error level (*p* < 0.01). This result confirms the one-dimensionality of the selected markers. So, the selected markers for measuring the study constructs were chosen correctly and accurately measure that component.

**Table 3:** The fit results of measurement models.

Constructs	Measurement item	Original TPB			Extended TPB		
		ƛ	t	Reliability and validity	ƛ	t	Reliability and validity
Intention	Int1	0.857	54.363	AVE: 0.658 CR: 0.889 α: 0.834	0.844	49.079	AVE: 0.658 CR: 0.890 α: 0.834
	Int2	0.867	55.355		0.868	61.580	
	Int3	0.733	26.303		0.733	23.990	
	Int4	0.808	38.144		0.820	47.293	
Attitude	Att1	0.804	35.293	AVE: 0.656 CR: 0.884 α: 0.827	0.804	38.016	AVE: 0.656 CR: 0.884 α: 0.827
	Att2	0.855	42.541		0.855	40.790	
	Att3	0.788	26.845		0.788	25.967	
	Att4	0.792	25.566		0.792	26.632	
SN	SN1	0.770	25.281	AVE: 0.573 CR: 0.843 α: 0.754	0.771	25.186	AVE: 0.573 CR: 0.843 α: 0.754
	SN2	0.738	25.131		0.736	23.508	
	SN3	0.734	23.047		0.734	23.369	
	SN4	0.784	36.334		0.786	36.734	
PBC	PBC1	0.694	16.625	AVE: 0.562 CR: 0.836 α:0.747	0.694	17.209	AVE: 0.563 CR: 0.836 α: 0.747
	PBC2	0.834	37.185		0.834	37.558	
	PBC3	0.669	15.531		0.670	15.006	
	PBC4	0.790	37.984		0.789	39.576	
RP	RP1	-	-	-	0.658	14.227	AVE: 0.504 CR: 0.811 α: 0.733
	RP2	-	-		0.692	18.155	
	RP3	-	-		0.571	11.669	
	RP4	-	-		0.731	19.387	
	RP5	-	-		0.742	40.314	
SBP	SBP1	-	-	-	0.748	22.906	AVE: 0.508 CR: 0.858 α: 0.802
	SBP2	-	-		0.769	27.980	
	SBP3	-	-		0.826	41.073	
	SBP4	-	-		0.703	19.586	
	SBP5	-	-		0.618	15.055	
	SBP6	-	-		0.574	11.661	

#### Reliability and Validity

In this step, CR, Cronbach’s alpha and AVE are investigated. According to the results presented in Table 3, it can be said that the CR of all the constructs in the proposed research model was higher than 0.60 and their Cronbach’s alpha coefficient was higher than 0.70. In addition, AVE for all the constructs of the proposed research model was more than 0.50; therefore, all the latent variables of the model had proper reliability and validity. This result means that the items selected to measure the constructs of the research were carefully selected and it provides the possibility of repeating the experiment.

#### Discriminant validity

Discriminant validity is possible when questions measuring one variable can be distinguished from questions measuring other variables. In terms of statistics, if the square root of AVE between the research variables is more than the correlation between them, it means that the research variables have appropriate discriminant validity (Fornell and Larcker, 1981). Based on the results presented in Table 4; it was observed that square root of AVE for the research constructs (0.71 < AVE < 0.92) was greater than the correlation between them (0.33 < r < 0.55). This result indicating the confirmation of the discriminant validity of the constructs in the proposed research model.

**Table 4:** Examining the discriminant validity of the research constructs.

Constructs	1	2	3	4	5	6
1-Attitude	0.81^a^					
2-Intention	0.72^**^	0.82^a^				
3-PBC	0.56^**^	0.72^**^	0.75^a^			
4-RP	0.63^**^	0.78^**^	0.61^**^	0.69^a^		
5-SBP	0.49^**^	0.62^**^	0.51^**^	0.53^**^	0.72^a^	
6-SN	0.71^**^	0.75^**^	0.67^**^	0.65^**^	0.47^**^	0.76^a^

a The square roots of AVE estimate.

** Correlation is significant at the < 0.01 level.

#### Evaluation of the research structural model

Various indices are used for investigating the fit of the research structural model (Table 5). Based on the proposed values of the presented indices and the reported values, it can be said that the model has a good fit and can be used to test the research hypotheses.

**Table 5:** Summary of goodness of fit indices for the measurement model.

Fit index	SRMR	D-G1	D-G2	NFI	RMS-Theta
Suggested Value	<0.1	>0.05	>0.05	>0.90	≤0.12
Original TPB	0.08	0.528	0.711	0.99	0.09
Extended TPB	0.06	0.627	0.728	0.99	0.08

**Figure 4:**
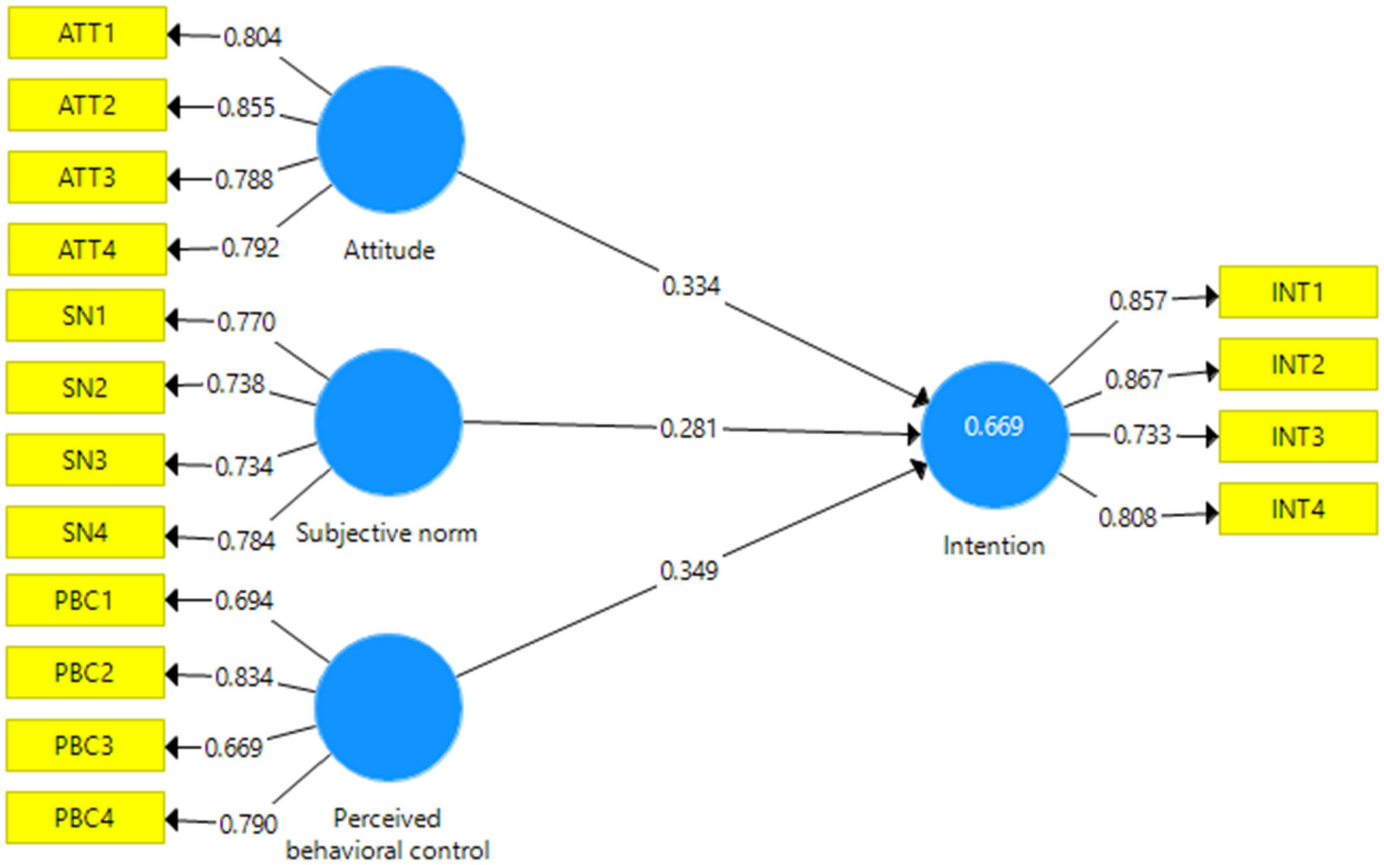
Original TPB structural model with standardized path coefficients.

**Figure 5:**
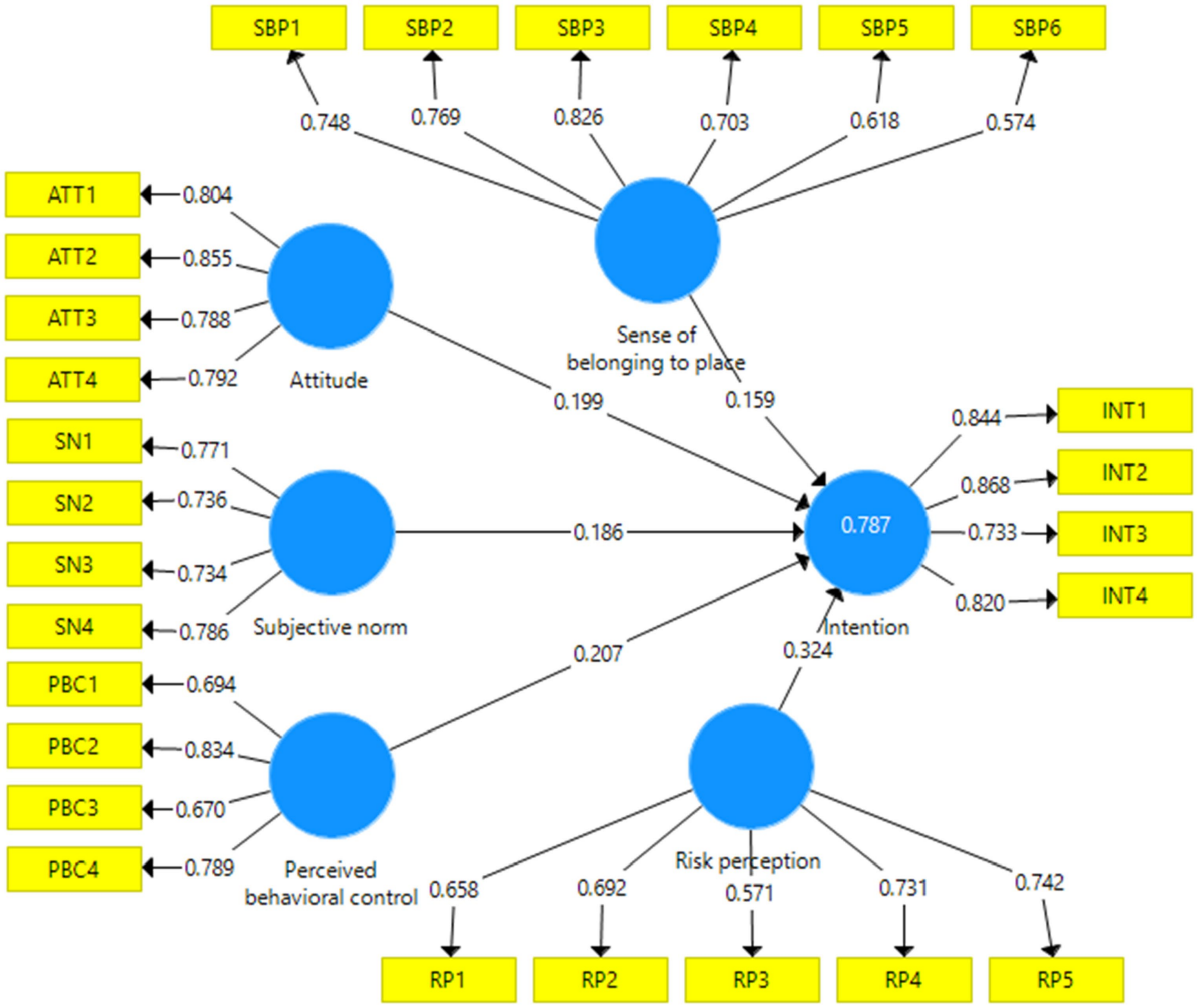
Extended TPB structural model with standardized path coefficients.

After confirming the measurement and structural models of the research using CFA, the path analysis method (structural model evaluation) was used to test the hypotheses in the form of the proposed research conceptual model. The model of the research path with standardized factor loadings has been presented in Figures 4, 5.

#### Test of the research hypotheses

In this step, the results of the final effect of the variables on the use of behavioral intentions were presented. The bootstrapping method was used to evaluate the significance of the path coefficient or beta. We used bootstrapping on 100 and 300 samples. The findings indicated that there was no change in the significance of the parameters in both cases, and the results had strong validity, because the significance of the relationships between variables was not affected by the sample size, and the only change that was made was in the value of the t statistic. Therefore, hypotheses can be tested in the form of regression model. The results showed that the extended TPB explained 78.7% of the behavioral intentions of local communities, in other words, the extended model has 11.8% higher explanatory power than the original TPB (Table 6).

**Table 6:** Results of the research structural models.

**Hypothesis**	Original TPB			Extend TPB		
	γ	*t*	Result	γ	*t*	Result
H1: Attitude → Intention	0.334	5.793	Confirm	0.199	3.510	Confirm
H2: SN → Intention	0.281	4.796	Confirm	0.186	3.110	Confirm
H3: PBC → Intention	0.349	7.770	Confirm	0.207	4.377	Confirm
H4: RP → Intention	-	-	Confirm	0.324	7.661	Confirm
H5: SBP → Intention	-	-	Confirm	0.159	3.821	Confirm

## Discussion

This study was conducted with the aim of investigating the intentions of local communities towards the protection of Arasbaran forests in Iran. In this research, a social-psychological model was used. Therefore, this research had two general goals: (i) investigating and the effectiveness of TPB in relation to the intentions of local communities to protect the forest and (ii) improving the explanatory power of TPB by adding two variables of RP and SBP. The general findings showed that the extended model has a higher explanatory power compared to the original one in explaining the behavioral intentions of people. In other words, the inclusion of two variables of RP and SBP is more effective than the original TPB, and a positive change in RP and SBP can have a significant effect on the behavioral intention of locals.

The results indicated that the model used in the context of behavioral intentions towards forest protection was very successful and unique, because the figure of variance explained by extended TPB was 78.7%. In the analysis of this result, it can be said that using the TPB model in the field of forest protection compared to the results of other studies on different issues such as food waste (Soorani and Ahmadvand, 2019), organic agriculture (Yazdanpanah and Forouzani, 2015), water saving (Savari et al., 2022a), etc. has been more successful because the amount of explained variance in present study is higher than them. Another importance of TPB in investigating forest conservation behavior was that all its variables became significant while in many studies on other fields (Yazdanpanah and Forouzani, 2015; Soorani and Ahmadvand, 2019; Savari et al., 2022a) all the variables were not significant, which indicates the good fit of the TPB model in explaining the behavioral intention of forest protection. The findings of this study provide new insights for policy makers to protect the environment and natural resources. These results can also be used in other regions of the world that are facing with the problem of forest destruction. In this research, SEM was used for testing the research hypotheses. In the following, the results are presented in order based on the research hypotheses.

The results of hypothesis (1) show that the attitude has a significant effect on the behavioral intention of locals. Studies of Popa et al. (2019), Tam (2020), Ullah et al. (2021), Sánchez et al. (2018) and Primmer and Karppinen (2010) support the findings of this section. In the analysis of this results, it can be said that researchers have always mentioned attitude as the key to understanding behavior and called it the most important influencing variable in explaining behavioral intentions (Zhong et al., 2019), because attitude refers to positive and negative evaluation from a behavior (Sánchez et al., 2018). Therefore, individuals who have a favorable and sustainability attitude towards the environment are always more inclined than other people not to leave destructive behavior in their surroundings environment (Savari and Gharechaee, 2020). Although it seems that the expected financial benefits resulting from a conservation behavior is important for the person, but the results of the studies show that social motives are more effective than financial ones in motivating people to appear environmentally-friendly behaviors (Savari et al., 2022b). Therefore, changing people’s attitudes through social activities and training courses in line with forest protection activities can be effective in this regard, because until people are unaware of the importance of an appropriate behavior and its consequences, there will be no change in their attitudes and beliefs.

The results of this research show that SN has a significant effect on the behavioral intentions of locals for protecting the forests. This finding confirms the second hypothesis of the research. This is consistent with the results of Gao et al. (2017), Sánchez et al. (2018) and Liang et al. (2018). The finding of this section indicates that individual’s behavior is influenced by different people in the society, and as a result of their influence or pressure, a behavior is performed or not. In other words, a person bases his intention on the basis of others’ wishes (Savari et al., 2022a), because SN refers to perceived social pressure to engage or not engage in a behavior (Liang et al., 2018). Therefore, if the behavior of forest protection is accepted by people with higher social position, it is also effective in the implementation of protection behavior by other individuals (Savari et al., 2022b). In this regard, it is suggested that individuals who have a higher social position among rural people should be used as communication channels with the residents in order to facilitate the implementation of forest conservation.

The findings of the test of hypothesis (3) show the significant effect of PBC on the behavioral intention of locals. This result is similar to the studies of Chee (2012), Sánchez et al. (2018) and Empidi and Emang (2021). Researchers always state that environmental conditions such as economic factors can affect the probability of using a certain behavior (Mehri et al., 2011). However, environmental factors are not able to create a sustainable behavior in the environment, but psychological factors can be more useful in this regard (Savari et al., 2022a). Based on this, it can be said that a higher level of psychological factors such as PBC among indigenous people can probably lead to forest preservation behavior. Because PBC plays an important role in the motivation and belief of a person for performing an activity (Savari and Gharechaee, 2020) and refers to a person’s confidence in his abilities to successfully perform a specific task (Asakereh and Yousofi, 2018). The importance of PBC variable among locals is due to the fact that many of them have little knowledge and awareness about how to protect the forest, so if their awareness about the effects of the forest on livelihood, income and environmental services is improved, they will apply protection behaviors and prevent destructive ones (Vahedi et al., 2016). Due to the importance of PBC in the intention to conservation behavior, it is possible to provide manuals of protective behavior to households and use lectures and educational programs along with them. These measures can improve the skills and knowledge of local communities in the field of forest.

The results of hypothesis (4) showed the significant effect of RP on the behavioral intentions of villagers to protect the forest. The results of Megías-Robles et al. (2022), Li et al. (2021) and Savari and Gharechaee (2020) support this finding. In the analysis of this finding, it can be stated that the RP of beneficiaries and people who have a close relationship with all types of natural resources and forests is a very important factor and its importance has been emphasized in various studies (Mumpower et al., 2016; Sabzehei et al., 2016). RP is a subjective evaluation of the nature of a threat and its severity (Azadi et al., 2019). In other words, RP is a mental construct and it deals with the issue of how much people worry about the risk of the activity they are doing or think that this action will cause trouble in the future (Arbuckle et al., 2015). This result indicates that if people seriously evaluate the risks and negative consequences of forest destruction and believe that these damages may have harmful effects on their livelihood in the future, the probability of performing forest protection behavior among them will be higher (Altman and Low, 1992; Chen and Tung, 2014). Therefore, by providing the statistics of forest destruction and its negative effects on environmental services and the livelihood of rural communities, it is possible to create a high RP in local communities and ultimately strengthened their motivation for protecting the forests.

Finally, the last investigated hypothesis (H5) showed the effect of the SBP on behavioral intention. The studies of Le Dang et al. (2014), Savari et al. (2021), Elias et al. (2018) and Zwiers et al. (2018) confirm this finding. In the analysis of this obtained result, it can be concluded that people’s belonging to a specific place is one of the ways that connects them to a society. The weakness of such intentions and social bonds makes a person feel free to commit crimes and abnormal actions (Milligan, 1998; Morse and Mudgett, 2017). SBP is developed through the growth of positive interaction of people and their social adaptation in that place, and the strength of this attachment is directly related to the amount of these connections (Mesch and Manor, 1998). In fact, people preserve the environment because of their SBP to it (Le Dang et al., 2014). Usually, people who have high SBP will not perform behaviors contrary to the norm of the place (Morse and Mudgett, 2017). Therefore, if the local communities have a high emotional connection with the forest, they will always try to protect it. As a result, residents’ SBP is one of the main reasons for the sustainability of settlements and natural resources (Hernandez et al., 2010; Ramezanpour et al., 2014).

## Conclusions and policy implication

The general aim of this research was to investigate the intention of local communities to participate in the protection of Arasbaran forests. The findings of the research indicated that the locals do not have a high intention for conserving the forests and their attitude in this regard are not favorable, which can lead to further destruction of these areas. In this study, to investigate the factors affecting the intention of local communities, extended TPB was utilized, which was able to explain nearly 80% of the behavioral intentions of people. The results of this research emphasize the importance of two variables of RP and PBC on the behavioral intentions of indigenous communities in the preservation of Arasbaran forests.

In general, the results of this research can greatly help policymakers in guiding local communities to behave safely and friendly with natural forests. Given that RP and PBC were the strongest predictor of locals’ behavioral intentions, therefore, it is necessary to conduct training programs and inform people about the positive consequences of forest protection. On the other hand, less use and reduced pressure on forests may lead to a decrease in the income of indigenous people, so it is better for the government and policymakers to reduce their dependence on the forest by diversifying employment and compensate their income reduction. In addition, due to the great importance of SN in the behavioral intention of rural people, it is possible to create a social pressure for protecting the forest using an appropriate culturalization. Finally, the last important policy that can help forest conservation is the development of NGOs and local forest protection organizations. People should be encouraged to become members of these organizations so that they can show appropriate behavior in times of crisis and emergency. Considering that some parts of these forests are destroyed every year due to fire, rural organizations can play a vital role in solving this problem.

In general, it is suggested that researchers pay attention to the following in their future studies: (1) Investigating the actual behaviors of forest protection should be considered because behavioral intentions are a necessary condition and a strong explanatory for actual behavior, but they cannot represent actual behavior, (2) It is better to pay attention to other psychological theories so that their explanatory power can be compared with TPB.

## Data availability statement

The original contributions presented in the study are included in the article/supplementary material, further inquiries can be directed to the corresponding author.

## Author contributions

All authors contributed to the study conception and design. Material preparation, data collection, and analysis were performed by MS and BK. The first draft of the manuscript was written by MS. All authors commented on the previous versions of the manuscript. All authors contributed to the article and approved the submitted version.

## Funding

The current paper is adapted from a research assigned in Agricultural Sciences and Natural Resources University of Khuzestan, with a Grant Number of 1401.32, and financially supported by the university; thereby we declare our appreciation for their help.

## Conflict of interest

The authors declare that the research was conducted in the absence of any commercial or financial relationships that could be construed as a potential conflict of interest.

## Publisher’s note

All claims expressed in this article are solely those of the authors and do not necessarily represent those of their affiliated organizations, or those of the publisher, the editors and the reviewers. Any product that may be evaluated in this article, or claim that may be made by its manufacturer, is not guaranteed or endorsed by the publisher.
